# Association of Delay in Breast Cancer Diagnosis With Survival in Addis Ababa, Ethiopia: A Prospective Cohort Study

**DOI:** 10.1200/GO.23.00148

**Published:** 2023-11-22

**Authors:** Alem Gebremariam, Adamu Addissie, Alemayehu Worku, Nebiyu Dereje, Mathewos Assefa, Eva Johanna Kantelhardt, Ahmedin Jemal

**Affiliations:** ^1^ Department of Public Health, College of Medicine and Health Sciences, Adigrat University, Adigrat, Ethiopia; ^2^ Global Health Working Group, Martin-Luther-University, Halle-Wittenberg Halle, Germany; ^3^ Department of Preventive Medicine, School of Public Health, College of Health Sciences, Addis Ababa University, Addis Ababa, Ethiopia; ^4^ School of Public Health, Wachemo University, Hosanna, Ethiopia; ^5^ Department of Radiotherapy Center, School of Medicine, Addis Ababa University, Addis Ababa, Ethiopia; ^6^ Surveillance and Health Services Research, American Cancer Society, Atlanta, GA

## Abstract

**PURPOSE:**

There are limited data on the association between delay in breast cancer diagnosis after breast symptom recognition and survival, particularly in sub-Saharan Africa. The recently launched Global Breast Cancer Initiative by WHO includes measuring delay as the core indicator for quality of breast cancer care. Herein, we examined the association between delay in breast cancer diagnosis with overall survival among women in Addis Ababa, Ethiopia.

**MATERIALS AND METHODS:**

A total of 439 women diagnosed with breast cancer from January 1, 2017, to June 30, 2018, in Addis Ababa were followed for survival to the end of 2019. Survival rates were estimated using the Kaplan-Meier method. The association between delay in diagnosis (>3 months after symptom recognition) and overall survival was computed using the multivariable Cox regression model after adjusting for demographic and clinical factors.

**RESULTS:**

Nearly 70% (303/439) of women with breast cancer were delayed in diagnosis of their cancer. During a median follow-up period of 25.1 months, 2-year overall survival rate was 73.5% (95% CI, 68.0 to 78.2) in women with diagnosis delay compared with 79.1% (95% CI, 71.2 to 85.1) in those women without diagnosis delay. In the multivariable Cox regression model, the risk of death was 73% higher (hazard ratio, 1.73; 95% CI, 1.09 to 2.74) in women with diagnosis delay compared with those without diagnosis delay.

**CONCLUSION:**

Delay in diagnostic confirmation of breast cancer after recognition of breast symptoms was negatively associated with overall survival in Addis Ababa, Ethiopia, underscoring the need to increase awareness about the importance of prompt presentation for clinical evaluation and referral for diagnostic confirmation to mitigate the undue high burden of the disease.

## Teaser

In Ethiopia, delayed diagnosis of breast cancer increases the risk of death, emphasizing the need for prompt diagnosis.

## INTRODUCTION

Breast cancer is the most commonly diagnosed cancer in women worldwide^1,2^ and it is the leading cause of cancer death in women in Ethiopia and other parts of Africa^1,3^ because of late diagnosis and lack of standard treatments.^4,5^ The survival rate is lower in resource-constrained regions such as sub-Saharan Africa than in wealthy nations.^2,6^ Among economically developing nations, the majority of breast cancer deaths^4^ happen among young women who are raising families and taking care of other family members. For instance, Ayele et al^4^ discovered that Ethiopia's median breast cancer mortality age was 37 years.

**CONTEXT**

**Key Objective**
Is diagnosis delay inversely associated with the survival of women with breast cancer?
**Knowledge Generated**
Only three in four women with breast cancer survived 2 years after their diagnosis. There is a strong correlation between a delayed diagnosis and a higher chance of death for women with breast cancer.
**Relevance**
The findings highlight the importance of prompt health seeking after the discovery of breast symptoms, prompt referral to diagnostic facilities by health care professionals, and the expansion of breast cancer diagnostic and treatment facilities in Ethiopia to lessen the disease's excessively high burden of morbidity and mortality.

Previous studies in Ethiopia and many other African countries reported a long delay in diagnosis of the disease after recognition of symptoms.^7-9^ For instance, a recent study found that in Addis Ababa, Ethiopia, approximately 73.3% and 43.8% of women with breast cancer received diagnostic confirmation after 3 months and 6 months, respectively, after the discovery of symptoms.^7^ Likewise, McKenzie et al^5^ reported that 71% of patients with breast cancer in sub-Saharan African countries were diagnosed 3 months or longer after recognition of symptoms. This delay likely reflects both patient and health system factors.^5,10^ Length of diagnosis delay is a key indicator of quality of breast cancer care, according to the recently established Global Breast Cancer Initiative by WHO.^11^ However, it is unclear whether delay in diagnosis (from symptom recognition to diagnosis) of breast cancer is associated with survival in sub-Saharan African settings.

Previous studies from other parts of the world on the association of delay in breast cancer diagnosis with survival considered delay as patient^12,13^ or health care provider delay,^12,14,15^ and none of these studies found an association except the study by Richards et al,^13^ which associated patient delay with better survival. The inconsistent findings between these studies may reflect differences in methodologic approaches in assessing the association, including lack of uniformity in the definition of delays and cutpoints.^16^ Specifically, most of the studies assessed the effect of either patient delay or health care delay on survival, although the underlying causes of patient delay and diagnostic delay are intertwined,^17^ and it is difficult to partition the independent contribution of each to survival. Also, most of the studies addressing the relationship between patient or diagnosis delay and survival were based on retrospective data, which are prone to data incompleteness and recall bias for dates of symptom(s) recognition, first medical consultation, and diagnostic confirmation. Herein, using prospectively recruited women with breast cancer in Addis Ababa, Ethiopia, we examined the association between overall survival and diagnosis delay (waiting >3 months from the date of breast symptom recognition to the date of diagnostic confirmation). We chose to use diagnostic delay (patient delay and health care provider delay combined) instead of patient delay and health care provider delay separately because dates of first medical consultation are subject to recall bias as a substantial proportion of women diagnosed with breast cancer in Addis Ababa visit several health care facilities before diagnostic confirmation.^18^ A similar approach of combining patient and provider intervals into symptom interval (duration) was used in examining the association between delay in diagnosis and late-stage breast cancer in parts of Africa^5,19^ because their causes are linked.^17^

## MATERIALS AND METHODS

This study was part of a larger study on the experience of women with breast cancer in Addis Ababa, Ethiopia, whose protocol was published previously.^20^ Briefly, a total of 441 women diagnosed with primary invasive breast cancer from January 2017 to June 2018 were recruited prospectively from seven major health facilities in Addis Ababa, Ethiopia, representing approximately 90% of breast cancer cases recorded in the Addis Ababa population-based cancer registry during the corresponding period. The study was ethically approved by the Institutional Review Board (018/17/SPH) of the College of Health Science of Addis Ababa University, and verbal consent was obtained from the study participants.

A structured baseline questionnaire about health behaviors, breast symptoms, and diagnosis was administered to study participants by trained interviewers.^20^ Participants were followed every 6 months for vital status (death, main outcome of the study) by calling their personal or family phone numbers. At least three calls were made throughout the day, evening, and on weekends to enhance response rates to minimize loss to follow-up. Of the 441 women, two were excluded from the study as they dropped within a month of follow-up. In addition, another two women were lost to follow-up after 16 months and 17 months of follow-up, respectively. These women were censored for 1-year survival, but they were considered lost to follow-up for the 2-year survival estimates. Overall survival was computed in months from diagnosis to death from all causes.^21-23^

The primary exposure variable was diagnosis interval (symptom interval), which is the time interval from date of recognition of first symptom(s) to date of breast cancer diagnosis.^5^ The date of first symptom recognition was obtained from face-to-face interviews with the women during recruitment, while the date of diagnosis was obtained from the patients' medical charts. Patients were considered delayed for diagnosis if the diagnosis interval exceeded 3 months.^24^ Age at diagnosis, marital status, level of education, occupation, monthly family income, and use of traditional medicine were collected during the in-person interview. Clinical explanatory variables such as stage, comorbidities, surgical treatment, radiation therapy, and hormonal therapy were extracted from medical records by senior oncology residents at about a year after diagnosis and end of follow-up. Two-year overall survival rates for groups of women with or without delay were calculated using the Kaplan-Meier method, and the survival rates were compared using the log-rank test.^25^ Hazard ratio for women with versus without diagnosis delay was calculated using a multivariable Cox proportional hazards model by adjusting for the variables with *P* value of <.25 in the bivariate analysis, including age at diagnosis, marital status, level of education, monthly family income, stage at diagnosis, surgery received, and hormonal therapy initiated. The graphical approach and goodness-of-fit test were used to verify that the proportional hazards assumption was met. A *P* value of <.05 was used to determine statistical significance.

## RESULTS

Table 1 shows sociodemographic and clinical characteristics of the study participants according to diagnosis delay. The mean age was 44.4 years (standard deviation = 12.2 years). Of the 439 women included in the final analysis, 69.0% (n = 303) were delayed, diagnosed >90 days after recognition of symptoms. Women delayed for diagnosis were more likely to be less educated, unmarried, use traditional medicine, and present with advanced-stage disease (Table 1).

**TABLE 1:**
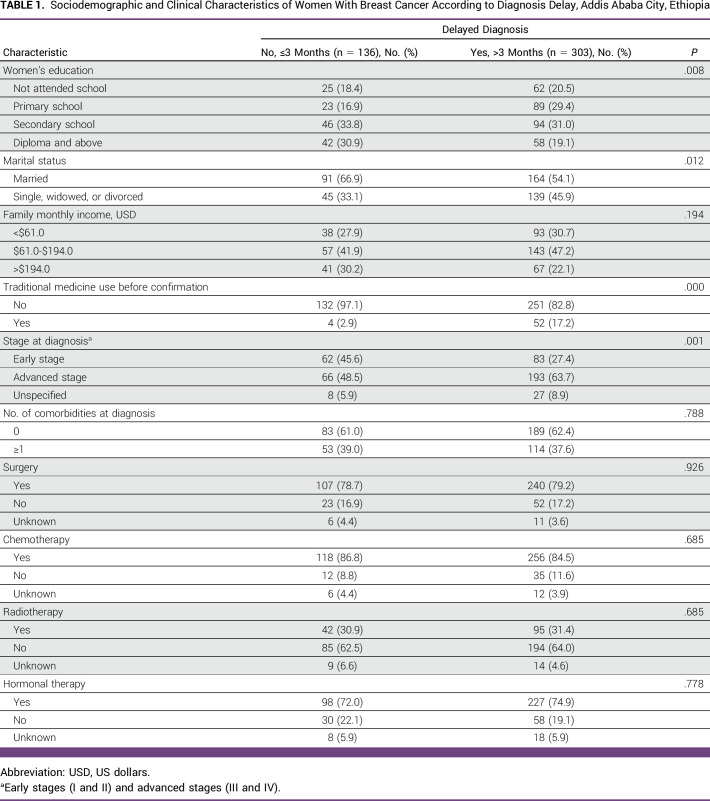
Sociodemographic and Clinical Characteristics of Women With Breast Cancer According to Diagnosis Delay, Addis Ababa City, Ethiopia

**FIG 1:**
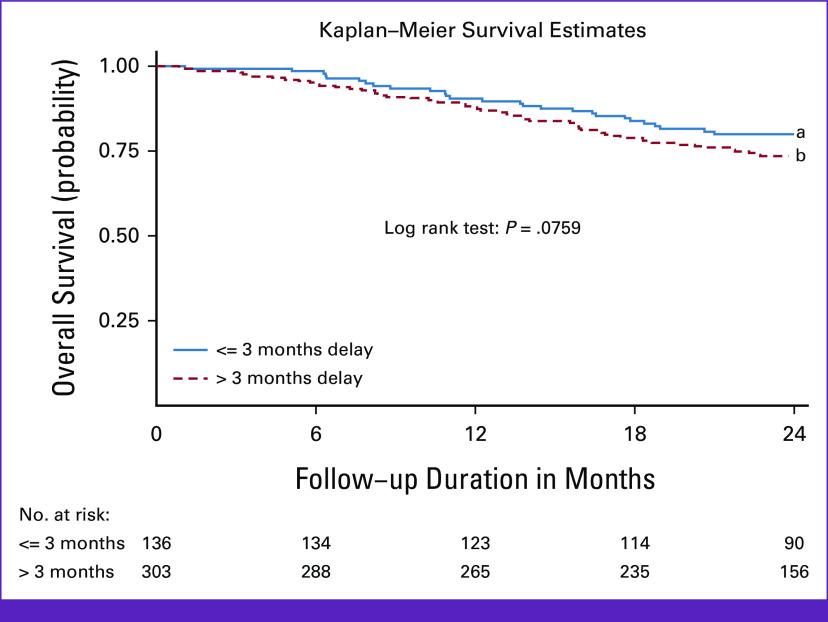
Overall survival rate of women with breast cancer diagnosed from January 1, 2017, to June 30, 2018, by symptom interval in Addis Ababa city, Ethiopia. The blue solid line (a) represents the survival of women who receive a diagnosis of breast cancer within 3 months of recognizing their first symptom(s), while the red short dashed line represents the percentage of women who survive after receiving a diagnosis 3 months after noticing their first symptom(s).

The median follow-up time, calculated from the diagnosis date, was 25.1 months (IQR, 19.3-31.4). Overall survival rate was substantially lower in women with diagnosis delay than in those without diagnosis delay (Fig 1). For example, 2-year overall survival rate was 73.5% (95% CI, 68.0 to 78.2) in women with diagnosis delay compared with 79.1% (95% CI, 71.2 to 85.1) in women without diagnosis delay.

After adjusting for stage at diagnosis and other potential confounders in Table 2, women with diagnosis delay had a 73% greater mortality risk than those without the diagnosis delay (hazard ratio [HR], 1.73; 95% CI, 1.09 to 2.74; Table 2). However, HRs for delays of 3-6 months and >6 months were generally similar.

**TABLE 2:**
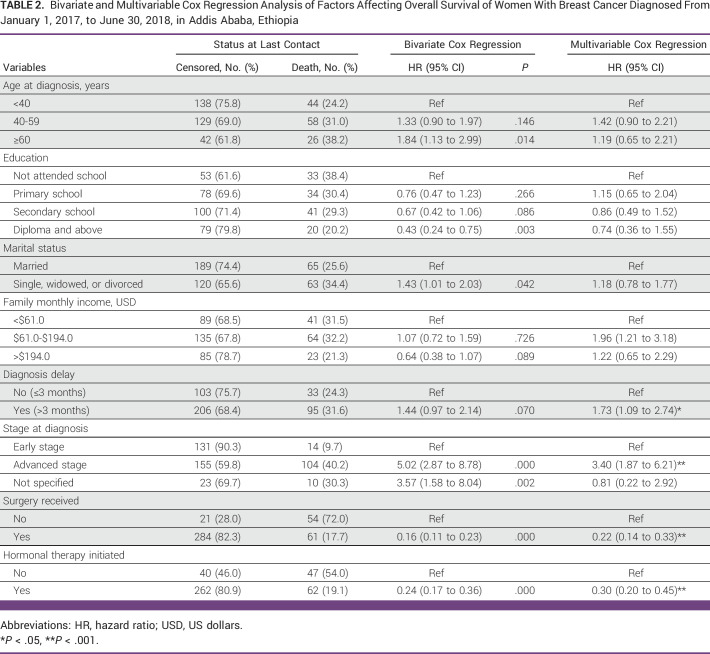
Bivariate and Multivariable Cox Regression Analysis of Factors Affecting Overall Survival of Women With Breast Cancer Diagnosed From January 1, 2017, to June 30, 2018, in Addis Ababa, Ethiopia

## DISCUSSION

On the basis of data from women with breast cancer recruited from seven major health facilities in Addis Ababa, covering approximately 90% of all newly diagnosed breast cancer cases in the city, we found that delay in diagnosis of breast cancer (diagnosis confirmation longer than 3 months after symptom recognition) was significantly associated with poor overall survival. This finding underscores the need for resource-stratified tailored interventions to shorten the time interval between symptom recognition and diagnosis to improve the survival of women with breast cancer in Addis Ababa and other parts of Ethiopia. Such interventions may include educating women, including young women, about breast health and the importance of prompt medical consultation after breast symptoms, as well as educating frontline health care providers about breast cancer symptoms and the significance of prompt referral for timely diagnosis of suspected breast cancer. Also, it is important to engage community and religious leaders and breast cancer survivors on this topic.

Our finding of a nearly two-fold higher risk of death in women with more than 3 months' delay for medical consultation after recognition of breast symptoms, compared with those women with breast cancer without such delay, cannot be compared with findings from previous studies. Previous studies on the association of breast cancer diagnosis delay with survival were based on patient delay (time interval from recognition of symptoms to medical consultation)^12,13^ or health care provider delay (time interval from date of patient medical consultation to diagnostic confirmation).^14,15^ None of these studies found an association between provider or patient delay with survival except the study by Richards et al,^13^ which associated patient delay with better overall survival and contrary to what was expected. Richard et al^13^ speculated that those delayed breast cancer cases may have been biologically less aggressive. However, Unger-Saldana et al^17^ argued that fundamental causes of patient delay and health care provider delay are linked; thus, it is challenging to disentangle the individual contributions of each to survival.

Our finding of 2-year overall survival rate (75.2%) was considerably higher than that reported in rural parts of Ethiopia (53%).^26^ Reasons for the elevated survival rate in Addis Ababa include a higher proportion of early-stage breast cancer diagnoses (35.7% vs 15%)^7^ and better access to care. Nevertheless, majority of breast cancer in Addis Ababa and other parts of Ethiopia are diagnosed at advanced stage of the disease, underscoring the need for interventions to downstage the diseases. These interventions include programs to increase breast cancer awareness both in the general public and in frontline health care providers. According to a recent study, nearly half of the women in Addis Ababa were unaware of breast cancer.^27^ Also, a third of patients with breast cancer in our sample had never heard of the disease before diagnosis and 92% of them attributed their first symptoms to a simple breast swelling or to other noncancer issues.^18^ A survey of female nurses in Addis Ababa showed that 42% of the nurses had inadequate general knowledge about breast cancer.^28^

A strength of our study is the use of prospective data to show an association between diagnostic delay and overall survival of women diagnosed with breast cancer. However, our study has several limitations. First, we were not able to examine the association between diagnostic delay and cause-specific survival because of lack of death registration in the city. However, since our follow-up period for vital status was relatively short (median of 25 months), the majority of deaths was likely to be from breast cancer. Second, dates of symptom recognitions were subject to recall bias. But, our study only included women who had recently received a breast cancer diagnosis. We also used local events such as festivals and holidays to recall the date of first symptom recognition and clinical presentation. Third, loss to follow-up is a major issue in cohort studies. However, we mitigated the influence of this on our estimates by using multiple contact information. Of note, only two participants were lost to follow-up.

In conclusion, in a cohort study with prospectively enrolled women diagnosed with breast cancer in Addis Ababa, we found that delay in breast cancer diagnosis was associated with poor overall survival and only three in four women survived 2 years after their diagnosis. The finding reinforces the need to enhance public awareness about breast cancer and early and continuous education of women about the importance of prompt medical consultation after breast symptom recognition and referral for diagnostic confirmation to mitigate the undue high burden of the disease in the city and throughout the country. There is also a need to enhance health care infrastructure, especially diagnostic walk-in centers with rapid referral options in the country to improve breast cancer care and outcomes.

## Data Availability

The data that support the findings of our study are available from the first author (alemg25@gmail.com) upon reasonable request.
